# *It’s very difficult to set the boundaries, it’s human nature to want to respond*: exploring health professions educators’ responses to student mental health difficulties through a positioning theory lens

**DOI:** 10.1007/s10459-023-10254-7

**Published:** 2023-06-09

**Authors:** Debra L Marais

**Affiliations:** Research and Internationalisation Development and Support, Faculty of Medicine and Health Sciences, Stellenbosch University, Cape Town, South Africa

**Keywords:** Boundaries, Care ethics, Health professions educators, Positioning theory, Professional development, Student mental health, Student support

## Abstract

By virtue of their teaching role and contact with students, health professions (HP) educators are often the first point of connection for students who are experiencing mental health difficulties. Educators are increasingly expected to include some form of pastoral care in their role. Mental health-related interactions with students may have a negative emotional impact on educators, particularly when roles and expectations are not clearly defined and where boundaries are not managed effectively. Using positioning theory as a lens, this study explored how educators experienced such interactions and how this manifested in positions, storylines, and speech acts. Interviews were conducted with 27 HP educators at a faculty of medicine and health sciences. Reflexive thematic analysis using inductive coding identified themes corresponding to the nearing, weighted, ambivalent, and distancing positions participants adopted in relation to students with mental health difficulties. There was fluidity in and between positions, and more than one position could be occupied simultaneously; participants each moved through different positions in response to different relational situations. Multiple storylines informed these positions, representing how moral- and care-informed responsibility intersected with responsiveness to make certain actions possible or impossible. Normative and personal value narratives were evident in storylines, in many cases underscored by care or justice ethics. The value of positioning theory in facilitating reflective faculty development initiatives for educators engaged in these interactions is discussed.

## Introduction

The prevalence of mental health disorders among university students is well recognised (Bantjes, et al., 2016; Storrie, et al., 2010). Health professional students seem to be an especially at-risk group (Hope & Henderson, 2014; Macauley, et al., 2018). Symptoms of mental illness negatively impact on students’ academic performance and increase the likelihood of dropout (Hartrey, et al., 2017; Storrie, et al., 2010). Universities need to be equipped with the necessary support, both formal and informal, to prevent and treat mental disorders. Health professions (HP) educators are often the first connection point for students with mental health difficulties (Quinn, et al., 2009). While this includes requests for academic concessions (e.g. extending deadlines) (Becker, et al., 2002), research suggests that these encounters increasingly involve provision of emotional support to distressed students, whether initiated by the staff member or the student (Hartrey, et al., 2017; Hughes, et al., 2018).

Student-educator interactions around mental health issues can be affirming and supportive (Brockelman & Scheyett, 2015). However, educators providing such support frequently report not receiving any training in dealing with mental health difficulties (Hughes, et al., 2018; Margrove, et al., 2014). Academic staff feel ill-equipped to assist these students (Hughes, et al., 2018; Quinn, et al., 2009) or have negative attitudes influencing their willingness to assist (Becker, et al., 2002; Gulliver, et al., 2019). In addition, more approachable staff may have more students accessing them, increasing the emotional demands on these staff. This frequently goes unrecognised (Hughes, et al., 2018). Staff may also struggle to balance support for students with other obligations of their role (Brewster, et al., 2022) which, together with increasing workload demands on staff (Barkhuizen & Rothmann, 2008; Kinman & Jones, 2003), may result in mental health challenges for staff themselves (Margrove, et al., 2014).

Academic staff are increasingly expected to include some form of care work in their role (Hodgson & Bretherton, 2021; Laws & Fiedler, 2012; Samuel & Konopasky, 2021). Caring may be part of how educators construct their professional identities (Avis & Bathmaker, 2004) and HP educators in particular tend to identify with this trait (Hughes & Byrom, 2019). Care work is consistent with calls for an ethics of care in academic spaces (Bozalek, et al., 2014; Sykes & Gachago, 2018), where care is not just a disposition but a moral imperative (Thompson, 2015). However, care work is not only undervalued and marginalised, but may require educators to do further ‘work’ to manage their emotions in responding appropriately (Hawkins, 2019; Isenbarger & Zembylas, 2006). Additionally, Tronto’s ethical conceptualisation of ‘good care’ as involving attentiveness, responsibility, competence, and responsiveness is difficult to enact in practice due to resource constraints and conflict between needs (Evans & Thomas, 2009; Tronto, 1993). An ethics of care also assumes that educators understand boundaries and have an awareness of their own dynamics in order to enact care responsibly, both for self and other (Hawk, 2017).

HP educators must juggle multiple roles and responsibilities (Laws & Fiedler, 2012; Stoddard & Borges, 2016), and supporting students with mental health difficulties can impact these roles (Spear, et al., 2021). In particular, these educators must juggle their responsibility to their students and their duty to protect patients and the profession into which students are graduating, creating dilemmas about when to be responsive, and to whom (Hughes, et al., 2018; Spear, et al., 2021). Challenges in maintaining the ambiguous boundaries between these roles and responsibilities, as well as between the personal and professional, are commonly reported (Hughes & Byrom, 2019; Spear, et al., 2021), with educators concerned about either overstepping boundaries or not doing enough (Hughes, et al., 2018; Payne, 2022). Navigating interpersonal closeness and distance in this way is a form of boundary work (Chreim, et al., 2013) requiring educators to invest additional time, effort, and emotional resources in their student interactions. Mental health-related interactions in particular may therefore have a negative emotional impact on educators, particularly when roles and expectations are not clearly defined and where boundaries are not managed effectively (Hughes, et al., 2018).

The multiple roles forming medical educators’ professional identities (Harden & Crosby, 2000; Stoddard & Borges, 2016) have often been presented as discrete and static. This fails to acknowledge the permeability of boundaries around these roles, which often evokes emotional discomfort (Browne, 2019; Laketa & Côte, 2022) and influences how individuals make sense of what positions and responses are – or are not – available to them in relation to another (Paulus, et al., 2009). In relational contexts, emotional boundaries can create distance or connection between self and other (Hayward & Tuckey, 2011; Mols & Pridmore, 2020). Interacting with students with mental health difficulties is one such relational context. In South Africa, as is the case in many other countries, student mental health has become a research focus (Bantjes, et al., 2016, 2019; Van der Walt, et al., 2020). However, less is known about how HP educators manage student mental health challenges and the role, responsibility, and boundary tensions outlined above. Using positioning theory, this study aimed to address this gap.

## Conceptual framework: positioning theory

Positioning theory (Harré, et al., 2009) was adopted in this study to explore meaning-making in the relational context of interactions with students with mental health difficulties and how this affected educators. The theory focuses on interpersonal encounters, whereby “people are positioned or position themselves with respect to rights and duties to act within evolving storylines, and on the basis of claims about relevant personal attributes” (Harré, et al., 2009, p. 5). Positions are more fluid than roles and are guided by storylines, the “evolving internal narratives that actors see themselves playing out” (Hu, et al., 2019, p. 709). These narratives include both social-cultural or normative narratives and personal narratives, which discursively construct micro-level identities in social interactions (Kayi-Aydar, 2021), linking to work that explores the narrative construction of professional identity (Rees, et al., 2019; Watson, 2007; Watson & Mcluckie, 2020). The taking up of these positions is always in relational context (Andreouli, 2010), with positions being manifested in actions – or ‘speech acts’ (Alcock & Bellugi, 2018; Hu, et al., 2019).

Positions and storylines both enable and limit what an individual feels capable of doing in any given social interaction (Bourgeois-Law, et al., 2020). Notably, while different storylines may operate in parallel, they may also conflict, causing tensions between positions (Hu, et al., 2019). The dynamic nature of positions, and the emphasis on individuals’ construction of meaning and what positions storylines make possible (Bourgeois-Law, et al., 2020; Hu, et al., 2019), aligns with the above discussion on how educators navigate positions and boundaries in their interactions with students. Stolle et al. (2018, p. 100) suggest that positioning theory could help educators to “resist boundaries” that limit them. In addition, in positioning theory, positions centre on moral orders about rights and responsibilities which, in turn, influence action (Harré, 2012; McVee, 2011). HP educators not only come up against relational boundary dilemmas but must navigate multiple, sometimes conflicting moral responsibilities (Hughes & Byrom, 2019). Previous work has shown, for example, how medical educators shifted between an ethics of justice and ethics of care in navigating what they perceived to be moral responsibilities in interpersonal situations (Green & Gruppuso, 2017; Hu, et al., 2019). This work suggests that HP educators shift between positions depending on the situation, rather than drawing boundaries between one or the other moral imperative. This fluidity in positions and moral responsiveness to the particularities of the interaction is consistent with positioning theory.

Previous research has used positioning theory to explore identity (Andreouli, 2010; Sargeant, et al., 2017; Vanassche & Kelchtermans, 2014), role and boundary conflict (Bourgeois-Law, et al., 2020; Stolle, et al., 2018), and an ethic of care (Gkonou & Miller, 2017). Positioning theory has been previously applied in HP education research (Matthews, et al., 2020; Sargeant, et al., 2017). Hu et al. (2019) adopted positioning theory as a lens to show how medical educators positioned themselves in student support encounters, alternatively embodying either an ethic of justice or care (Green & Gruppuso, 2017). However, in their study, support was framed in general terms as part of medical educators’ roles, and no studies could be found that used positioning theory to explore the experiences of HP educators responding to students with mental health difficulties.

Positioning theory was thus employed as “theory-informing [iterative] inductive analysis”, where the theory helps tell a story about the data (Varpio, et al., 2020, p. 992). The theory provided a lens to illuminate how educators took up various positions in their interactions with students with mental health difficulties and how they navigated boundaries around and within positions during these encounters. This study thus used positioning theory at the intersection of roles, boundaries, and care in the relational interactions between educators and students. This has the potential to expand on notions of ‘boundary work’ to better understand how educators’ narratives about their roles, responsibilities, and rights in such encounters position them in ways that shape how they respond. Faculty development initiatives focusing on how HP educators navigate these positions and the storylines that inform them could facilitate greater self-awareness and flexibility in managing these relational interactions involving mental health challenges.

## Methods

This descriptive, cross-sectional study addressed the research question: How do HP educators experience interactions with students who present with mental health difficulties at the Faculty of Medicine and Health Sciences (FMHS) at Stellenbosch University (SU)? Because the aim was to explore how educators constructed meaning in their accounts of these experiences, the study was located in an interpretivist paradigm and used qualitative methodology. Although positioning theory was developed for the exploration of social interactions, it has also been applied to interview and narrative data to understand how participants construct themselves and others in their narrative responses (Gkonou & Miller, 2017; Kayi-Aydar, 2021).

All academic, clinical, administrative, and support staff employed at the institution who have at least monthly direct (contact) or indirect (virtual) interaction with students (undergraduate and/or postgraduate) in the performance of their duties were invited to participate. Of those who responded to an initial call, I purposively selected a sample representative across as many environments as possible. Participants were selected in an attempt to achieve representation of both male and female HP educators, as well as of the medical and health sciences programmes.

Demographic information collected included gender, age, position in faculty, length of time working at faculty, frequency of direct interaction with undergraduate and postgraduate students, and nature of direct interaction with students. Twenty-seven semi-structured interviews were conducted. Of the 27 interview participants, 16 were women and 11 men. All participants were in frequent contact with undergraduate and/or postgraduate students and reported experiences with students with mental health difficulties. All were HP educators in a range of positions: clinical facilitator, lecturer, senior lecturer, associate professor, and professor. Ages ranged from 35 to 65 years, with roughly equal representation across age groups. Duration of participants’ employment at the faculty was between one and 30 years.

Interview questions were developed based on previous research (Hughes, et al., 2018; McAllister, et al., 2014); broadly, they asked participants to describe one or more of their experiences with students with mental health difficulties. I began each interview asking participants to think of and describe any specific examples of interactions with students with mental health difficulties. I was interested in and listening for particular aspects of participants’ experiences – how they managed such interactions, whether they felt they had the capacity and support, their perspectives of students with mental health difficulties and mental health in general, what their own affective experiences were in navigating these interactions, and how they managed their different roles as health professions educators in supporting these students. I would allow the participant to speak freely but would pick up on and ask for elaboration where these aspects were mentioned. Because I was interested in participants’ storylines, I would follow their narrative trajectory, allowing the space for detailed elaboration and going down ‘tangents’ with participants by encouraging them to speak more about any aspect of their experience that they raised.

Interviews were scheduled at a time convenient to each participant and took 45–60 min. I conducted all interviews in English; they were audio-recorded and later transcribed for analysis. I employed a transcriber but conducted all data analysis myself. Participants were assigned pseudonyms to protect confidentiality. Data collection was completed just before the COVID-19 lockdown in March 2020.

Interview data were analysed in Atlas.ti using reflexive thematic analysis, following Braun and Clarke’s guidelines (Braun, et al., 2022). Thematic analysis involves a recursive process of coding and analysis, whereby themes are built on initial codes and represent a higher level of patterned reflection of meaning within the data set (Braun & Clarke, 2006). I first engaged in a soft-focus, inductive coding, engaging in depth and iteratively with the data identifying patterns of ideas, most notably the issue of boundaries in various manifestations. Participants appeared to move closer to or further away from students in their interactions and encountered tensions between different felt responsibilities. This prompted a return to the literature to identify a conceptual framework that may be useful in guiding further inductive analysis (Varpio, et al., 2020). The final themes were informed by iteratively developed inductive codes, with positioning theory as a frame for engagement with the data. Throughout the analysis, I engaged in discussions with critical friends about the developing codes and themes.

Reflexivity is a central criterion for ensuring quality in qualitative research (Frambach, et al., 2013). Before beginning analysis, I wrote about my preconceptions and ideas regarding this topic, reflecting on my position in relation to the topic and participants in this study: as a researcher, as psychologist, as fellow employee, and as interviewer in relation to my participants. In my role at my institution, I have frequent interactions with students but few in which mental health comes up. I have once *been* the student with mental health difficulties. Throughout the study, I have been reflective of how and where these positions place me in relation to my data and the meanings I have interpreted from them. I was aware of the social interactions taking place between the interviewees and I and the positions and internal narratives that may be playing out, particularly as employees at the same faculty. Although this may have influenced the extent to which participants felt free to discuss issues that arose for them in relation to the topic, I found the opposite to be the case: interviewees seemed eager to talk about the challenges they experienced, and many expressed how important they felt this research was.

Ethics permission was obtained (reference: N19/07/085). Participants were informed that participation was voluntary and that they could choose not to answer any questions or withdraw from the study at any time, after which they signed an informed consent form. All reasonable measures were taken to protect participants’ confidentiality. Participants were informed of the limits to protection of confidentiality in qualitative studies.

## Findings

This section describes the themes and sub-themes that were identified during the inductive analysis.

### Themes (positions) and sub-themes (storylines)

Themes came to represent positions that participants held in relation to encounters with students with mental health difficulties and comprised nearing, weighted, ambivalent, and distancing positions. Positions inform and are informed by storylines; sub-themes identified were thus seen as the storylines guiding each themed position (Table 1). Participants’ responses, then, were speech acts in which their positions and storylines manifested. All positions could be informed by one storyline or by multiple storylines held concurrently. The storyline sub-themes are integrated throughout the text within each theme section, highlighted in boldface.

**Table 1:** Themes and sub-themes

Themes (positions)	Sub-themes (storylines)
Nearing positions	• Staff as supporter • Staff as parental figure/caretaker • Staff as provider • Staff as friend/confidante • Staff as fellow sufferer
Weighted positions	• Feeling ill-equipped • Feeling responsible • Feeling heavy • Feeling for students
Ambivalent positions	• Education/student • Patients/student • Other students/student • Parents/student • Confidentiality/disclosure • Helping/harming • Professional/personal
Distancing positions	• Staff should keep their distance • Staff must protect the profession/patients • Students will behave badly/take advantage • Staff are intolerant of mental health issues • Staff are expected to be resilient/invulnerable

It is important to note that the positions and storylines were not intended to imply judgment and were seen to have neither positive nor negative valence. For instance, distance is sometimes required and appropriate as a way of enforcing necessary boundaries, whereas in some situations reaching out to students may resonate with how participants see themselves and their work as HP educators. In addition, although positioning theory is interested in how individuals position both themselves *and others*, I have not focused in this study on the ways in which students were positioned by the positions that educators occupied. However, some of this was implicit in the storylines identified and is highlighted below. I chose to use the word “staff” in the themes to distinguish between staff member and student; staff represents the HP educators who participated in the study.

### Nearing positions

Positions through which participants drew closer to students and/or mental health issues form the nearing positions theme. This positioned staff as having a moral or emotional responsibility to respond. The storylines enabling this position relate to how staff saw themselves and the care they could offer to students. Students were positioned as vulnerable and in need of care.

For the storyline about **staff as supporter**, this support took different forms, each drawing staff closer to students who might be struggling. In some cases, this was through supportive acts like “*just checking up on*” individual students (Participant S); in other cases, educators offered support as mentors to students, facilitating their learning to care for themselves so that they could care for others “*as part of becoming a health professional”* (Participant T). Occasionally, participants spoke about their belief that they should advocate for struggling students: “*There wasn’t any question in my mind…that my role was still to advocate for her, and to be a voice if she couldn’t be her own voice at that stage”* (Participant G).

The position of drawing near to students with mental health difficulties was made possible in some instances by the **staff as parental figure/caretaker** storyline: “*I get to that point where I tell them, listen, I don’t mind going to drop you [at rehab]…And then when you go drop them there, then you feel like you are dropping off your own child”* (Participant V). Others took on the responsibility as caretaker of students, in the absence of family or formal mental health support, which included visiting students in hospital out of “*care [for] the student, you want there to be some sort of positive resolution. In both cases there, they didn’t have family nearby”* (Participant F).

Similar to the parent/caretaker storyline was the **staff as provider **storyline, which seemed to be compelled more by practicalities than emotion. This took the form of in-kind practical or financial support to students. Some staff took students “*to the shopping centre and paid for some groceries and stuff like that for him*” (Participant B), while others had students “*stay with me for a week or so, and then we tried to find a place for her to stay”* (Participant N). Interestingly, some staff explicitly recognised that they were crossing their own boundaries, while others did not perceive the assistance they provided as unusual.

Nearing positions were also informed by the **staff as friend/confidante** storyline. Most participants whose actions manifested this storyline saw this as a natural extension of their professional relationship with the students. One participant explicitly identified as seeing her postgraduate students as “*friends. I would like it to be that people are comfortable to actually chat to me…I don’t want to strictly just be like a colleague to someone”* (Participant S). Other staff felt comfortable interacting on a more casual basis with students, going for coffee, using emojis to communicate, and talking over Whatsapp:

*I said to her, but let’s meet for coffee…so we met at* [coffee shop] *and we had a nice chat…And afterwards, I had her on Whatsapp, and I’ve asked her, how are you doing? And, I’m thinking of you today, things like that.* (Participant A)

The last storyline was **staff as fellow sufferer**. Participants spoke not only of their own struggles with mental health but of the importance of sharing this with students as a way of helping them: “*I suffered from depression at one stage…So I can relate to that, and I’m not hesitant to tell the students that I’ve been through that thing myself”* (Participant J). Others emphasised the importance of being “*a bit vulnerable to make yourself appear to be human, because otherwise…they will not be open to come and speak to you about things”* (Participant N).

### Weighted positions

Weighted positions were those in which participants felt weighed down by their emotion and/or responsibility for students. However, ‘weighted’ is also used here to represent the importance or value attributed by participants to being responsive to students with mental health difficulties. In both instances, weighted positions were imbued with a moral duty to respond, despite the impact on the staff member.

Staff acted from weighted positions when they held the narrative of **feeling ill-equipped** to handle students or the situation. For some participants, this occurred when students presented with more severe symptoms, which “*was a bit of a big eye-opener, and I can’t deal with this, I don’t know how to deal with this”* (Participant C). Participants also sometimes felt that they were lacking sufficient knowledge or training to deal with the situation, thinking that “*I’ll deal with whatever happens, I don’t need to be trained, or whatever, until it happens with you, and then you realise, shucks, maybe I need a bit more preparation for this"* (Participant F). In other cases, participants felt ill-equipped approaching students: “*I don’t feel ever comfortable…it’s not an easy conversation to start*” (Participant L).

Participants also inhabited weighted positions when their storyline was one of **feeling responsible** for students struggling with mental health difficulties. This was expressed through worry that they may miss something important, “B*ecause I mean, I don’t want to be the one that says, okay, I overlooked this, ja”* (Participant D), or a worry about what they *had* done: “*I was still not quite sure if we did the right thing, but I’m also not sure what else I should have or could have done”* (Participant L). Some participants spoke about incidents where students had committed suicide, feeling responsible for not doing more to help.

*But how do you get over somebody who you didn’t help? You don’t, do you? So it’s always at the back of your mind and you just hope that you’ve learnt something from it, and next time, you will do something differently.* (Participant R)

Participants also acted from weighted positions when **feeling heavy** from helping students with mental health difficulties. Here, there was a sense of being burdened by their help for students. These “*heavy discussions take a toll*” (Participant C) and impacted on their own health or mental health:

*Dealing with these students is not easy; it’s complicated, and at times, it is frustrating, and it is emotionally draining. It’s part of the reason for my tiredness, now, is having to deal with these sorts of cases on quite a regular basis.* (Participant J)

Participants expressed the additional heaviness felt when they were unable to debrief about their experiences: “*I’ve had sleepless nights, where you worry that those students aren’t getting the help that they need, and they’re going to do something…it is difficult to sit with that information and that worry, and I can’t debrief”* (Participant Y).

When staff were weighted by **feeling for students**, they empathised with how students were feeling or felt concern that “*this is what they walk with…it’s really not nice, it’s not, really. I look at the kids and they are so young, and they are so burdened with issues”* (Participant W). Others felt for how students were “*finding the clinical environment in the hospital very negative and very harsh, and that worries me terribly”* (Participant B).

### Ambivalent positions

Ambivalent positions were where participants found themselves conflicted between different duties and responsibilities. Ambivalence is imbued with a sense of inertia, caught between students with mental health difficulties and other tasks or responsibilities, or between two opposing actions available to them. Students were positioned as requiring support but doing so created conflict with the requirements of someone or something else. This created conflict for staff about whether and how to respond.

For many participants, as HP educators, the conflicting **education/student** storyline informed the ambivalent position they occupied when interacting with students with mental health difficulties. They experienced tension between their responsibilities as educators and trying to assist or manage these students once they had disclosed their difficulties. Research supervisors found the boundaries of the supervision relationship increasingly difficult to manage with mentally unwell students: “*I referred a student to another supervisor…because it was a very, very emotionally needy student, and I just thought, okay, there’s a real need here but…I need to account for the* [thesis] *that doesn’t get done”* (Participant U).

Participants also spoke about challenges involved in evaluating student academic or clinical performance when they knew that students were struggling: “*So this whole problem of disclosure, that it compromises your objectivity as the person who, in the end, must assess the student, and on the clinical platform assess their fitness to practice”* (Participant O). The particular demands of training health professionals highlighted this frequently felt conflict:

*Where do you draw the line? You have to be tough on these guys, because you can’t have them operate on people if they don’t know what they’re doing, if they don’t have the ability to function under pressure. But at the same time, apply too much pressure and you break someone.* (Participant S)

Ambivalent positions were also manifested in the **patients/student** storyline, where participants had to juggle their responsibilities to patients and struggling students: “*If you’re not feeling good, you should address it, but in the background, we work with patients…I’m just very cautious as to, where is the line between our responsibility to patients*, [and] *our responsibility to our own mental care”* (Participant H). This storyline was also evident on a wider scale, where educators felt reluctant to “*allow students who can’t manage themselves onto the clinical platform. Because it does so much damage, potentially. Not just with individual clients but with systems”* (Participant T).

Some of the ambivalent positions occupied by staff were informed the **other students/student** storyline, feeling responsible for both the student with mental health difficulties and other students around them. One participant spoke of the conflict between wanting the best for the student as well as for the student community, but experienced this as a dilemma when the symptomatic student was “*not as discreet as you would have liked her to be. So this now becomes the problem of everyone who lives with her, which makes it my problem as well”* (Participant F). Participants also spoke about other students’ resentment when struggling students were absent or not pulling their weight, especially in a clinical setting:

*Other students start resenting this, where students will say, but that one always gets away with everything. Or I’ll say, okay, on a busy clinic…where is that one? The others would roll their eyes, could probably not get out of bed this morning, with the resentment, we do that, we manage to do this.* (Participant O)

Sometimes, the staff member felt obligated to involve the parents if they felt the student was unable to make sound judgements themselves. Mostly, however, staff were contacted by parents who were worried about or wanted to discuss their child. This was the **parents/student** storyline that informed an ambivalent position. This placed one participant in the position of hearing different versions of what was happening from the student and her parent when her mother phoned and

*asked me if I can go and have a look at her because she’s very worried about her. So I said that I just spoke to her, coincidentally, and that she’s fine. She said, ja, but I mustn’t be bluffed by that because her daughter is like that.* (Participant D).

Many other participants felt compromised if parents called them to ask about their child:

*A parent will phone you, and start, tutut*, [and] *you can’t say anything because you don’t know if the student gave consent. And so we’ve had to say to parents, I’m sorry, I cannot discuss this with you. I first need your child to give consent.* (Participant X)

Participant X also spoke about the ‘rule’ at the university that the student was the client, not the parents, and thus staff could not disclose anything to parents without student consent. This frequently manifested in ambivalent positions through the **confidentiality/disclosure** storyline. Sometimes, staff had been told by other students that someone was struggling and felt unsure about how to manage this:

*So with this student that I was talking about with the self-harm, for example, there’s how to approach that when I’m not really supposed to know about it. So there’s confidentiality involved, she’s got friends, they’ve told me…she’s got parents, should I tell her parents? But again, confidentiality.* (Participant F)

Staff who were frequently disclosed to by students found it difficult to hold the tension between creating a confidential space and knowing when they could – or should – disclose to others. Many participants also mentioned their frustration with not knowing whether students had taken up their referral to the Student Counselling Centre, again because of confidentiality.

*On the one hand, I feel that the fact that students are comfortable to come and talk to me...On the other hand, it’s very frustrating for me, because a lot of the time, they share confidential things with me that I have to sit with and not share it with colleagues…But I am sometimes very worried about students and I feel sort of helpless because, obviously, I don’t know if they go and seek the help that they should get, because that whole process is confidential.* (Participant Y)

Other participants spoke about decisions they made to disclose to colleagues, either to debrief or to check in with colleagues about whether they had done the right thing. This uncertainty often manifested in ambivalent positions through the **helping/harming** storyline, with participants speaking about the difficulty navigating “*that fine line between, it’s not really our job, and so do you take on more than you’re supposed to, and then what happens if something goes wrong?”* (Participant R). Others spoke about the conflict between responding with compassion and coming “*to a point where you maybe are not helping the situation, and a bit overstepping your role”* (Participant AA).

The final storyline informing participants’ ambivalent positions was the **professional/personal** conflict. For some, this was recognised as crossing a boundary without necessarily causing conflict:

*There have been cases where I’ve probably gone beyond what would be regarded as a strictly academic relationship…But I mean, isn’t that what makes us human? I’m not the sort of person who can just walk away from pain that people are experiencing.* (Participant Z)

One participant expressed ambivalence about being seen as an approachable person:

*And I do have a nickname in the one class, the Mother Hen. And I thought it was a bit of a compliment, in a way, but at the same time also, it was, okay, but I am an academic, you know, ja…* (Participant U)

In some cases, being the go-to person for students with difficulties came at personal and professional expense:

*So it can become a ‘go to speak to that one, he’ll be able to help you’…because there’s always the sucker that always does everything. And when you’re busy for an entire day and you’ve got nothing to show for it, except for two or three students that maybe are now still alive*. (Participant C)

This storyline was also evident in the boundaries that some staff tried to draw around their personal time and relationships, while still supporting students: “*So you try and assist and support somewhat, but, for me at least, I hold back a bit…at the end of the day, I’ve also got my time and my family, so you have to balance that”* (Participant F).

### Distancing positions

Staff occupying distancing positions created or maintained separation from the student with mental health difficulties, either intentionally or due to perceived normative expectations. This positioned staff as having a (moral) duty not to respond, with staff employing different storylines as to why this had to be the case. Students were positioned as somehow dangerous, bad, or mad, behaving unprofessionally or irresponsibly.

The storyline that **staff should keep their distance** saw distancing almost as a given; this could be expressed as though this was who they were, and alternatives were unthought or unknown: “*Some people are more motherly…I am more factual. I’ll try to find out what is the problem and act accordingly, refer the person if needed. I will never try, myself, to help them through their lives”* (Participant H). For other staff, the distancing was more indirect, where they employed ‘tools’ to guard against getting too close. These included faculty guidelines, lack of knowledge or qualifications, large student numbers, and making referrals to professional services: “*You have to actually draw a line, by identifying the referral places…So then you’re not getting the student off your radar, but there isn’t then personal involvement in their psychiatric condition* [and] *…their finance, and so on”* (Participant V). Other staff were very clear that supporting these students was not part of their job: *“I don’t see it as my role to support individual students on their journey to wellness…it’s outside my job description”* (Participant O).

Some staff could distance themselves using the storyline that **staff must protect the profession/patients**. In these cases, the staff themselves were the tools of protection between the students and patients/profession, thereby justifying their position of distance. Some participants emphasised patient safety, asserting that “*if they’re having problems, certain problems – not everybody – they actually need to be curtailed for their own health, as well as the patient’s safety, quite frankly”* (Participant R). Others referred to protection of their profession: “*I don’t know how to say this nicely, or empathetically, but there still is a* [professional] *standard that needs to be met…whether or not somebody’s dealing with a mental illness”* (Participant G).

Staff could adopt a distancing position through the storyline that **students will behave badly/take advantage**, possibly through some form of malingering. The speech acts of some participants suggested that students could not be trusted because of how they would take advantage of the system:

*I don’t really know what to do about it yet, or what to think about it…students are now taking mental health days, or personal days…And the other two had to carry the call and also write the test on the Friday. So…is there abuse of the system?* (Participant H)

One participant felt exasperated by how much additional work these students created, such as frequently having to schedule make-up tests for the same student/s, saying, “*I often feel that I didn’t sign up for this”* (Participant O).

Distance could also be created by the storyline that **staff are intolerant of mental health issues**, either themselves or others, in which case, the distancing position seemed to be occupied involuntarily. For Participant AA, there was ambiguity in her position, with lack of clarity regarding whether she included herself as part of the intolerant ‘people’: “*I think a lot of people still see mental health difficulties as a personal failure or a lack of character, and other people are coping, so why aren’t you?”* (Participant AA).

Some participants referenced stigma as underlying the intolerance. One spoke about her own difficulties with accepting mental illness:

*So as a professional, you might feel that I need to be accepting of all people, and there’s no stigma around mental illness. But in my personal life, I still flinch, maybe, when someone says Valkenberg* [psychiatric hospital]…*I still have issues with mental illness*. (Participant T)

The storyline that **staff are expected to be resilient/invulnerable** enabled some participants to occupy a distancing position from students and mental health issues. Some participants used this storyline to make sense of the distance adopted by other staff members:

*I mean, one of the worst things in medicine is this whole, well, we had it bad, therefore you must have it bad. And as soon as one does something to improve the life of students…*[then it’s] *oh, the students of today, they’re soft, and that kind of attitude…people forget struggles they had.* (Participant I)

Other participants believed that this was the perception held by students: “*even as a clinician that is qualified. Because if I burst into tears on the ward…students would see that as a weakness and shy away from it…They will not commend me for showing my feelings”* (Participant H). Or this was a perception held by some invisible ‘other’: “*Especially in this faculty of health, where we’re all supposed to be so wonderful and perfect, things are not always disclosed. And because you’re a doctor, and you’re a medical practitioner, you’re supposed to be okay”* (Participant X). Participants also felt that student mental health was prioritised by the faculty at the expense of staff mental health, which was a storyline employed to justify their own distance from student mental health.

## Discussion

This study explored HP educators’ experiences of interacting with students with mental health difficulties through a positioning theory lens. The variety of responses that such interactions evoke and the multiple roles that HP educators must navigate made positioning theory, with its emphasis on dynamic and fluid positions, a particularly useful conceptual framework. Participants in this study adopted nearing, weighted, ambivalent, and distancing positions, each informed by multiple storylines and manifested in their speech acts. Implicit in these positions were educators’ assumptions about their rights and duties in relation to students with mental health difficulties as well as about the students themselves; these influenced whether and how they responded.

From one perspective, the positions and storylines identified in this study could be seen as ways in which educators crossed over or stayed behind boundaries or were stuck between them. Much of the research on university staff interactions with students in need of support identifies the issue of boundaries as something that staff struggled with, suggesting that they need assistance either in making roles and boundaries clearer or in setting/maintaining boundaries (Payne, 2022; Vogan, et al., 2014). In practice, boundary work in relational interactions is more nuanced and complex (Hughes & Byrom, 2019; Hughes, et al., 2018; Mols & Pridmore, 2020). The implication of the notion of boundaries is that one either has them or one does not in any given situation, and that they are tangible and static, whereas a positioning theory lens allows for the fluidity of positions and their underlying storylines. This better resonates with the complexity of navigating interpersonal interactions, allowing for positioning closer to or further away from others depending on the storyline/s about rights and duties in the unfolding relational encounter.

Generally, all participants in this study moved through all positions, each with different storylines informing the positions in different situations (Bourgeois-Law, et al., 2020). Contrary to Hu and colleague’s (2019) findings, no dominant position emerged. In line with Hu, et al., however, there was fluidity in both the positions adopted and the storylines informing these positions, with more than one position being occupied simultaneously. In such cases, the storylines informing these positions were different but congruent – for example, being friend/confidante (nearing position) and feeling heavy (weighted position). The positions and storylines evoked depended at least in part on the relationship with the student and the context in which this was located, as well as how the student was ‘presenting’ with their mental health difficulties. One participant spoke of a strong belief that her supervisory role was to advocate for a depressed student in support of her studies; in a hospital context where a student was acting out unprofessionally, this participant asserted that such students should not be allowed to graduate as health professionals. In line with positioning theory, this foregrounds the importance of the relational context in which these interactions unfolded.

What stood out in the nearing and distancing positions is that the speech acts in the former were generally concerned with individual students, while the speech acts in the latter tended to be framed more abstractly, about students in general. As with Hu et al.’s participants, these positions seemed to embody ethics of care and justice (Green & Gruppuso, 2017) in the “moral space” of student support (Hu, et al., 2019, p. 707). When participants adopted distancing positions, the normative value assumptions in their storylines were concerned with procedural rules and regulations, fairness, and objectivity (Green & Gruppuso, 2017). In contrast, nearing position storylines prioritised the ethic of care values of empathy and responsiveness to individual circumstances (Green & Gruppuso, 2017). Consistent with previous research, participants shifted between these positions (Green & Gruppuso, 2017; Hu, et al., 2019), suggesting that different value systems about rights and duties were activated in different situations. Whether and how to respond was linked to participants’ storyline assumptions about what their moral responsibility was in relation to students with mental health difficulties.

Only one participant repeatedly emphasised her belief that assisting these students was not part of her job description; she moved primarily between distancing and ambivalent positions and most clearly embodied an ethic of justice. Perhaps by virtue of their willingness to participate in this study, all other participants identified with caring for students as part of their HP educator role. This care, however, was not without complexity or conflict, as evidenced by the four positions that educators moved between. Tronto’s care values of responsibility and responsiveness (Tronto, 1993), in particular, unfolded in different ways in participants’ perceived rights and duties with respect to the student, others, and themselves. As discussed below, in ambivalent and distancing positions, justice- and care-informed notions of responsibility were evident in participants’ storylines which in turn influenced their responses.

Participants’ storylines in this study thus represented different ways in which responsibility intersected with responsiveness to create moral orders (positions) that made certain actions possible or impossible. These actions played out as moving towards, moving away from, being stuck down, or being stuck in-between. Kayi-Aydar (2021) suggests that there are two kinds of storylines which are simultaneously activated: cultural/social narratives and in-the-moment narratives. Responsibility, in particular, is a moral obligation informed by implicit cultural/social norms and values (Evans & Thomas, 2009), which could include what is institutionally and professionally expected. In-the-moment narratives are guided by personal orientations and values (Hu, et al., 2019; Lönngren, et al., 2021). It is also likely that participants’ in-the-moment narratives in this study were influenced by how much they identified with care work as part of their professional identity and role (Andreouli, 2010) and with the cultural/social narratives around this. These storylines played out in participants’ perceived rights and duties, which influenced how they responded, whether as a movement (towards or away from) or in an inertia. Boundary work, then, became about taking up a position that then enabled or limited certain responses (Chreim, et al., 2013). In the same way that boundary work is nearly always simultaneously enabling and constraining (Slembrouck & Hall, 2014), when participants took up a position informed by a particular storyline, certain rights and duties were realised while others were precluded.

When adopting nearing positions, participants’ storylines espoused both responsibility and responsiveness. Tronto identified attentiveness, responsibility, responsiveness, and competence as dimensions of (an ethics of) care (Sykes & Gachago, 2018; Tronto, 1993). Participants in nearing positions seemed to embody these care values, with attentiveness and competence implicit in their responses. In general, there were no expressions of conflict in these nearing position responses, suggesting that participants’ personal narratives were in alignment with normative care narratives as well as, perhaps, with perceived institutional expectations of care work. Many have advocated for how an ethics of care could enhance teaching and learning in higher education (Bozalek, et al., 2014; Lynch, et al., 2020; Sykes & Gachago, 2018). A responsibility to respond in a caring way to students with mental health difficulties was intrinsic to how participants saw their professional role when they took up nearing positions (Hodgson & Bretherton, 2021; Samuel & Konopasky, 2021). How participants saw themselves (as carer) and how they saw the students (as in need of care) may have allowed for alignment between perceived personal rights and other-duties inherent in participants’ storylines. This, in turn, enabled a responsiveness that did not seem to require participants to engage in explicit boundary work to manage tensions.

Participants taking up weighted positions also felt a responsibility to be responsive to students’ mental health difficulties, but this weighed heavily on them – their felt duties seemed to come at a cost to their (personal) rights. This is consistent with evidence that managing this obligation takes its toll on educators (Hughes, et al., 2018; McAllister, et al., 2014) regardless of whether they nonetheless identify with care as part of their role, as suggested by the personal and normative narratives informing these storylines. Providing this support to students was a “labour of love” (Vogan, et al., 2014, p. 484). There was also an explicitly expressed lack of competence in the feeling ill-equipped storyline, as noted in other studies (Hu, et al., 2019; Hughes, et al., 2018). Perhaps implicit in this is participants’ expression of a right to feel equipped to be able to fulfil their duty to respond. The emotion present in the feeling heavy and feeling for students storylines supports the notion that to engage in such relational interactions with students is to “be undone” (Hawkins, 2019, p. 824). In this context, educators were likely to have regulated their own emotions in managing their self-presentation (Evans & Thomas, 2009) and engaged in boundary work to manage their closeness to or distance from the student.

When in ambivalent positions, participants seemed caught between responsibility and responsiveness. Their sense of responsibility was split between the student and other groups or concerns, complicating their capacity or willingness to respond. Implicit in some of the storylines in this position was a belief that students should be taking responsibility for themselves. In taking up ambivalent positions, educators occupied uncomfortable spaces between responsibility and responsiveness (Browne, 2019) and, in some cases, between an ethic of care and an ethic of justice in terms of their perceived rights and duties. There is discomfort in the liminal spaces where tensions cannot be reconciled (Laketa & Côte, 2022), requiring a greater degree of emotional boundary work by educators (Hayward & Tuckey, 2011). Boundary work entails determining what should be taken care of and what does not constitute care (Antoni, et al., 2020). These tensions in who and what should be responded to were underscored by tensions between perceived duties and obligations in this study. In the ambivalent position, with the exception of the personal/professional storyline, personal rights were overshadowed by these perceived responsibilities.

Intrinsic to distancing positions seemed to primarily be a moral responsibility *not* to respond to mental health difficulties displayed by students. Here, participants’ perceived rights and duties aligned less with care ethics and more with an ethics of justice, where storylines had an emphasis on rules, objectivity, and fairness, and participants tended to speak about students in abstract, general terms. In Vogan et al.’s (2014) terms, participants had switched from a labour of love to a labour of law, taking on the role of judge (Bourgeois-Law, et al., 2020; Green & Gruppuso, 2017). Here, participants also sometimes projected the responsibility onto students to follow acceptable professional and moral obligations, emphasising the autonomy and self-governance inherent in the ethics of justice (Botes, 1998). Storylines in the distancing position alternated between a duty to protect others (from students with mental health difficulties) and/or a right to protect themselves. The ‘othering’ of students with mental health in some storylines may also have been a form of boundary work that created distance and prioritised personal rights.

This study adds to the growing body of research using positioning theory in HP education (Hu, et al., 2019; Matthews, et al., 2020; Sargeant, et al., 2017). The findings of this study suggest that the boundary work done by HP educators to manage interactions with students with mental health difficulties could be usefully expanded to incorporate awareness of the positions and storylines informing how and where boundaries are placed, if at all. Positions, like boundaries, are inherently relational and navigating them is a normative and personal value-driven process, informed by implicit and explicit assumptions about rights and duties. Findings point to the ethics of care and ethics of justice inherent in the rights and duties of educators’ supportive interactions with students. Relational interactions, particularly those involving mental health difficulties, evoke moral responses that engender one or more storylines about the unfolding interaction and what response is required based on perceived rights and duties. These storylines position educators in various ways that enable or constrain action in relation to the other. This may be seen as a form of boundary work. However, positioning theory’s emphasis on fluidity between storylines and positions opens up more options for educators to move through than ‘setting boundaries’. Positioning theory may be a valuable framework for thinking through challenges faced by educators when interacting with students with mental health difficulties, helping them to become aware of the storylines informing their positions with respect to rights and duties and to recognise alternative storylines and positions available to them.

## Practice and research implications

Interactions with students with mental health difficulties is a reality for HP educators that should be incorporated in faculty development, moving beyond a focus on roles and boundaries. Positioning theory takes the conversation about HP educators ‘setting boundaries’ forward. Positioning theory-informed professional development (Hu, et al., 2019; Vanassche & Kelchtermans, 2014) expands on faculty development initiatives that focus only on increasing competence and confidence in boundary setting when dealing with mental health issues in students. While the availability of alternative positions and storylines is in itself complex, HP educator development using positioning theory has the potential to mitigate conflict experienced by facilitating meaning-making about the contradictions inherent in HP educators’ multiple roles and increasing awareness of alternative positions – and therefore responses – available (Bourgeois-Law, et al., 2020). Faculty development initiatives could facilitate collegial conversations that enhance intersubjective meaning-making and better understanding of underlying assumptions regarding rights and duties with respect to students with mental health difficulties, which may in turn engender more consistent responses to students experiencing difficulties (Haynes & Macleod-Johnstone, 2017; Hughes, et al., 2018; Paulus, et al., 2009). In examining and orientating the normative and personal values implicit in their responses, the ethics of justice and ethics of care in particular may be useful frameworks for reflection in faculty development (Bozalek, et al., 2014; Vanassche & Kelchtermans, 2014). Building on the ethics of care that many seemed to adopt, HP educators could be encouraged to incorporate self-care ethics in their professional practice. This includes greater self-awareness and an understanding of how sense is made of relational interactions, thereby fostering balance and resilience in higher education practices (Bryan & Blackman, 2019). Further, interventions focusing on educator wellbeing will inevitably impact the wellbeing of students (Abery & Shipman Gunson, 2016; Brewster, et al., 2022).

A gap left by this research is exploring how the positions adopted by educators positioned students in these interactions, and vice versa; research with students who have experienced mental health difficulties in university settings would be a useful avenue of inquiry to pursue. Action research using positioning theory as a reflective framework could enhance the practice-related recommendations above. It might also be interesting to explore the care and/or justice value-informed positions taken up by educators across different academic/professional fields, using positioning theory to build on Green and Gruppuso’s (2017) work. The emotional impact of mental health-related interactions on educators was not explored in this study, although participants were clearly moved in different ways by these. Future research could employ an explicit focus on the emotional nature of positions, storylines, and speech acts (Gkonou & Miller, 2017; Kayi-Aydar, 2021), particularly as it relates to support encounters around mental health issues. Integrating a psychoanalytic approach with positioning theory may be especially useful in exploring emotion (Nixon & Scullion, 2022).

## Limitations

The findings of this study are limited by the sample only including HP educators. Administrative and other support staff in frontline positions would likely have added texture and diversity to the findings. Similarly, the study was conducted in only one faculty in one country which may limit the transferability of the findings. However, international research suggests that the findings of this study have relevance beyond our local faculty (Bourgeois-Law, et al., 2020; Hu, et al., 2019), particularly research problematising the notion of boundary-setting in educator support for students with mental health difficulties (Hughes, et al., 2018). This study also did not include positioning of the other in the analysis, which limited the extent to which educators’ positions could be understood in relation to students’ positions. Similarly, how students positioned themselves would have enabled or constrained what responses were available to educators. Finally, engaging in the interview together would have meant that the researcher and participant were adopting positions in relation to one another that went unexamined.

## Conclusion

Positioning theory provides a framework for analysing speech acts to explore positions and storylines that both enable and limit how an individual enacts what they perceive as rights and duties in any given social interaction. Moving beyond boundary setting, this study revealed that HP educators adopt nearing, weighted, ambivalent, and distancing positions in relation to students with mental health difficulties. Findings suggest that normative and personal value orientations inform these positions, which in this study mostly centred on an ethics of care or ethics of justice at the intersection of responsibility and responsiveness. A positioning theory analysis may be enhanced by a focus on how care values are embodied in positions adopted in student support encounters. Professional development practices in HP education could expand on skills development and role clarification to encourage greater awareness of the multiple positions available to educators in support encounters, and the storylines informing these positions.
